# Monophasic Epithelial Synovial Sarcoma Accompanied by an Inverted Papilloma in the Sphenoid Sinus

**DOI:** 10.1155/2012/379720

**Published:** 2012-12-04

**Authors:** Xiaohua Jiang, Qi Huang, Jianguo Tang, Matthew R. Hoffman

**Affiliations:** ^1^Department of Otolaryngology-Head and Neck Surgery, Sir Run Run Shaw Hospital, Medical College of Zhejiang University, Zhejiang, Hangzhou 310016, China; ^2^Division of Otolaryngology-Head and Neck Surgery, Department of Surgery, University of Wisconsin School of Medicine and Public Health, Madison, WI 53705, USA

## Abstract

A 58-year-old man presented with a six-month history of intermittent blood-stained posterior nasal discharge. Five years ago, he had a three-week episode of fitful light headaches. Nasal ventilation, olfactory sensation, and facial sensation were normal; there were no ophthalmological complaints. Coronal computed tomography (CT) scans revealed soft masses in the bilateral sphenoid sinuses with bone absorption. The patient underwent bilateral functional endoscopic sinus surgery and resection of right nasal papillary masses. Papillary masses and mucosa in both sphenoid sinuses were also removed. The mass in the left sphenoid sinus was diagnosed as two separate entities, one being a primary monophasic epithelial synovial sarcoma and the other an inverted papilloma, while the mass in the right sphenoid sinus was an inverted papilloma. After surgery, the patient underwent radiotherapy and chemotherapy. At the 50-month follow-up visit, there were no signs of recurrence.

## 1. Introduction

Synovial sarcoma (SS) accounts for approximately 8% of all soft tissue sarcomas and is the fourth most common type of sarcoma. SS can occur anywhere in the body, but it most often occurs in the paraarticular areas of the lower extremities. The head and neck region is the second most common site of involvement, accounting for up to 10% of cases [1]. To our knowledge, there is one report in the biomedical literature describing primary monophasic fibrous synovial sarcoma (MFSS) in the sphenoid sinus [2]. We report the first case of primary monophasic epithelial synovial sarcoma (MESS) in the sphenoid sinus; this sarcoma was part of a mass in one cell of the sphenoid sinus which was comprised of two separate parts, one being the synovial sarcoma and the other being an inverted papilloma. This case report was approved by the institutional review board and ethics committee of the Sir Run Run Shaw Hospital.

## 2. Case Report

**Figure 1:**
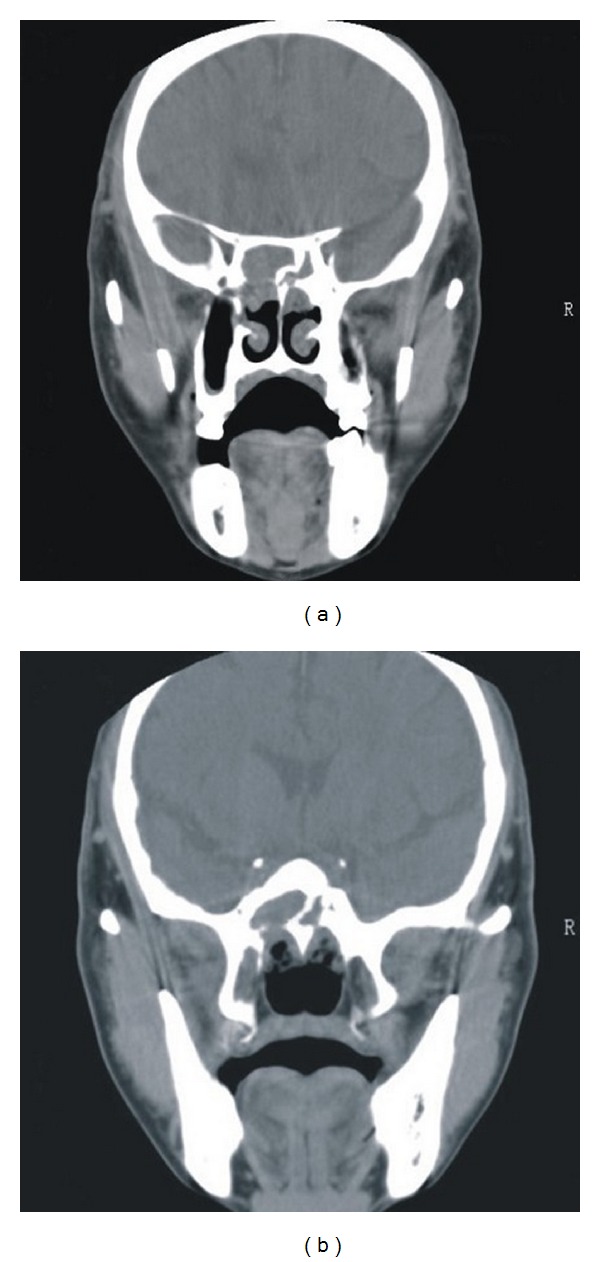
(a) Coronal computed tomography (CT) scan demonstrating edema in posterior ethmoid sinuses. (b) Coronal CT scan demonstrating soft masses in the bilateral sphenoid sinuses combined with bone absorption.

A 58-year-old Chinese man visited our department complaining of intermittent blood-stained posterior nasal discharge for six months and a three-week episode of fitful light headaches, which occurred five years ago. The patient reported normal nasal ventilation, olfactory sensation, and facial sensation. He had no ophthalmological complaints. Physical examination showed a polypoid mass in the left middle nasal meatus and a papillary mass in the right posterior olfactory cleft. No enlargement of the cervical lymph nodes was discovered. Coronal computed tomography (CT) scans of the sinuses demonstrated soft masses in the posterior ethmoid sinuses and bilateral sphenoid sinuses with bone absorption (Figure 1). Tissue biopsy under nasal endoscopy indicated that the left polypoid mass was a nasal polyp while the right papillary mass was an inverted papilloma. 

The patient underwent bilateral functional endoscopic sinus surgery and resection of the right nasal inverted papilloma. Intraoperatively, a polypoid mass was found in the left middle nasal meatus. There was mucus present in the posterior right and left ethmoid sinuses. No mass was discovered in the ethmoid sinuses; rather, only edema was present. Absence of tumor cells was confirmed by pathology. Papillary masses in bilateral sphenoid sinuses were also found. Radical excision of the papillary masses was performed. The mucosa of the sphenoid sinuses was also removed, while the sella turcica and lateral walls were kept intact. 

**Figure 2:**
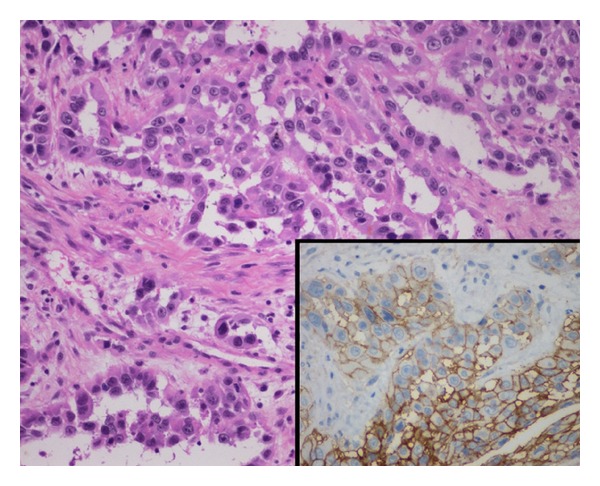
Atypical proliferate epithelial cells arranged like irregular glands (as in adenocarcinoma) and distributed among a fibrous substance. Atypical proliferate epithelial cells are immunoreactive for CD99 (insert). (Hematoxylin-eosin stain; original magnification ×200) (insert ×400).

**Figure 3:**
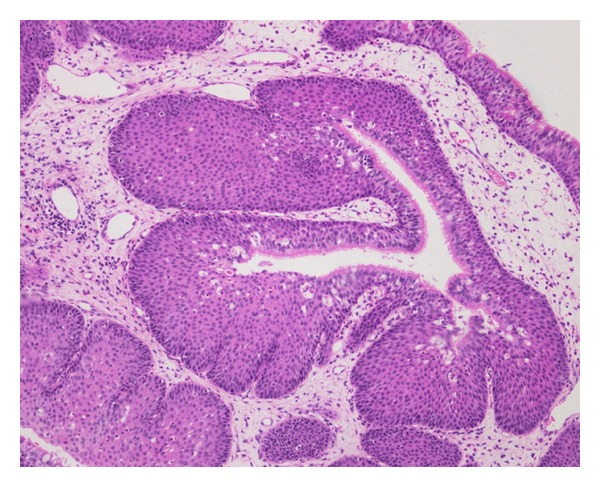
Papillary hyperplasia of the transitional epithelium. (Hematoxylin-eosin stain; original magnification ×100).

Pathologic examination with light microscopy revealed that the mass in the left sphenoid sinus actually consisted of two distinct parts. In one part, atypical proliferating epithelial cells arranged like irregular glands (adenocarcinoma-like) distributed among a fibrous substance were observed (Figure 2). In the second, papillary hyperplasia of the transitional epithelium was discovered (Figure 3). The mass in the right sphenoid sinus was also characterized by papillary hyperplasia of the transitional epithelium. The septum dividing the right and left sphenoid sinuses was intact, and there was no communication between the right and left masses; thus, we considered them to be separate entities. Immunohistochemical evaluation of the first part revealed positive staining for Vimentin and CD99 (Figure 2, insert) and positive staining focally for CK. It was negative for CD31 and Bcl-2. Three experienced pathologists independently made the final pathologic diagnosis of MESS accompanied by an inverted papilloma. Accordingly, the patient underwent adjuvant radiotherapy and subsequent chemotherapy beginning three weeks after surgery. At the 50-month follow-up visit, there were no signs of recurrence.

## 3. Discussion 

The incidence of SS in the paranasal sinuses is very low, particularly in the sphenoid sinus. To our knowledge, this is the first reported case of primary MESS with inverted papilloma in the sphenoid sinus. 

Pathologically, SS is a distinct sarcoma composed of two morphologically different cell types—epithelial and spindle cells. There are four subtypes of SS based on the relative proportions of the two cellular components and the degree of differentiation as follows: MFSS; MESS; biphasic; poorly differentiated [1]. Immunohistochemical confirmation is invaluable in the diagnosis of SS; however, there are still no specific or sensitive immunohistochemical markers. Generally, SS is immunoreactive for Vimentin, CK, EMA, S100, Bcl-2, CD99, and calponin and negative for CD34 and CD31 [1]. Detecting the chromosomal translocation t(X;18) which produces the chimeric gene, SYT-SSX, has been the key in diagnosing SS [3]. In this case, pathologic examination revealed an adenocarcinoma-like mass exhibiting expression of Vimentin and CD99 as well as focal expression of CK. These positive stains combined with the absence of CD31 supported the diagnosis, although cytogenetic analysis was not carried out.

The optimal treatment of SS is complete surgical resection with histologically clear margins; however, this cannot always be accomplished in head and neck cases because of the proximity of the tumor to vital structures [4]. Adjuvant radiotherapy and chemotherapy are also warranted. Knowledge regarding the prognosis of SS in the paranasal sinuses is limited due to a small number of cases, but some authors have suggested that the prognosis for patients with SS in the head and neck is better than for patients with SS in the trunk and extremities [5]. In this case, radical excision of the masses and sphenoid sinus mucosa provided clear margins, and adjuvant radiotherapy and chemotherapy likely cleared any residual disease as the patient was free of recurrence at 50-month followup.

Patients with SS in the sphenoid sinus do not have a specific presentation in prophase and are often free of signs or symptoms. Our patient only had intermittent blood-stained posterior nasal discharge and fitful light headaches that may not even have been caused by the SS. Once patients develop severe headaches, visual impairment, or restricted eye movement, the SS has involved surrounding structures. Early nasal endoscopy and computed tomography scans are essential; however, neither the radiologist nor the other consultants can be certain if a destructive lesion is SS, a different neoplasm, or inflammation. Thus, early tissue diagnosis is essential. Additionally, other problems arise if these pathologies occur concomitantly. Further work must be done to refine the diagnosis, prognosis, and treatment of synovial sarcoma in the paranasal sinuses.
